# Shifting patterns in emergency department attendance: a time series analysis

**DOI:** 10.1136/emermed-2024-214412

**Published:** 2025-12-04

**Authors:** Ahmad Alkhatib, Paul Aylin, Robert Klaber, Thomas Woodcock

**Affiliations:** 1 Department of Primary Care and Public Health, School of Public Health, Imperial College London, London, UK; 2 Department of Paediatrics, Imperial College Healthcare NHS Trust, London, UK

**Keywords:** emergency department, Multimorbidity, Routinely Collected Health Data, COVID-19, urgent care

## Abstract

**Background:**

Rising demand and limited capacity in primary care are often cited as reasons for the increasing pressure on emergency departments (EDs). The COVID-19 pandemic further strained but also reshaped healthcare services and their accessibility. However, an equally critical yet often overlooked factor is the increasing complexity of cases. This study assessed ED attendance trends for the Northwest London (NWL) population between February 2017 and September 2023, before, during and after the pandemic lockdown measures (March 2020–March 2021) in the UK as a whole and across sociodemographic and multimorbidity profiles.

**Method:**

We used the Whole System Integrated Care data warehouse in NWL. We conducted a segmented time-series quasi-Poisson regression for weekly ED attendance for two periods, before and after the pandemic lockdown measures, adjusting for seasonality and autocorrelation. We stratified the model by age, sex and quintiles of the 2019 Index of Multiple Deprivation. We analysed ED attendance trends by multimorbidity groups.

**Results:**

There were 3 365 279 ED attendances from February 2017 to September 2023. Before the pandemic, there was a statistically significant annual growth rate of 3.4% (rate ratio (RR) 1.034; CI 1.026 to 1.042), with a rising trend in attendance observed in all patient groups. After the pandemic, the overall trend stabilised (RR 1.002; CI 0.993 to 1.009). However, attendances have continued to rise for older age groups (61–75 years and 76+ years) and have increased for patients with multimorbidity and complex multimorbidity. Meanwhile, attendance has declined for the least deprived. For the other patient groups, attendance has plateaued.

**Conclusion:**

Following the pandemic, total ED attendances stabilised, but have continued to rise for older people, particularly those requiring complex care. This has implications for hospital capacity and places an increased strain on urgent and emergency care. We used high-quality representative population-level linked patient records data. The study was observational with limited causality. Further research should explore specific reasons behind these changes.

**WHAT IS ALREADY KNOWN ON THIS TOPIC**

- Pressures on emergency departments (EDs) are rising, and studies have shown increasing patient wait times.
- The COVID-19 pandemic further strained healthcare services and enforced substantial changes to healthcare service delivery, access and demand.
- It is unclear whether rising pressures and longer waits are due to more patient attendances or higher complexity of care.

**WHAT THIS STUDY ADDS**

- We found a trend of increasing ED attendances in England before the COVID-19 pandemic, and a decrease during the COVID-19 lockdown period. However, attendance plateaued between the easing of lockdown measures in March 2021 and September 2023.
- Following the COVID-19 pandemic lockdown, the proportions of older people and patients with multimorbidity and complex multimorbidity increased in EDs, while there has been a decline in attendance for younger people and for the least deprived.

**HOW THIS STUDY MIGHT AFFECT RESEARCH, PRACTICE OR POLICY**

- Case complexity is an important indicator of pressures on EDs and should be considered by the health system.
- There may be an opportunity to better manage ED pressures through understanding the causes behind shifts in case mix.

## Introduction

 The UK National Health Service (NHS) has been subject to escalating concerns voiced by professionals, patients, organisations and policymakers, regarding the system’s mounting pressures.^1^,^3^ In England, for example, the percentage of patients attending emergency departments (EDs) who are seen, treated and admitted or discharged within 4 hours declined consistently between 2013 and 2020.^3^ This is despite NHS England mandating that 95% of attendances should meet this target. According to data from February 2023, 10% of patients who attended ED waited more than 12 hours to be treated.^4^ ED crowding has a negative impact on patient outcomes, especially among those already experiencing health inequalities.^5^ Crowding has adverse consequences on the NHS, impairing responsiveness and resource utilisation and increasing staff stress and shortages,^6 7^ which feed into the cycle of increasing pressures.

The commonly cited reasons behind these pressures and delays in EDs include rising attendance volumes and limited capacity in primary care.^3 6^ However, an equally critical yet often overlooked factor is the increasing complexity of cases presenting to EDs. Anecdotal accounts and some emerging evidence suggest that ED patients are becoming older with more complex healthcare needs and longer stays.^5 6 8 9^ However, there is limited evidence from population-level studies. The evidence gap has become more pressing in the wake of the COVID-19 pandemic. It has profoundly impacted the NHS, altering needs and behaviours and access to healthcare and accelerating the restructuring of health services.^10^,^13^ There remains a lack of high-quality data to fully understand the longer-term shifts in ED case-mix.^14 15^

Our objective was to investigate trends of all-cause ED attendance, overall and broken down by demographic and chronic health profiles and accounting for the COVID-19 pandemic lockdown period in Northwest London (NWL) between February 2017 and September 2023. Our study aims to shed light on case-mix shifts as an important factor in understanding ED pressures and to inform research and policies aimed at improving health services in the post-pandemic recovery.

## Methods

### Setting and data source

We used linked retrospective longitudinal data from the Whole System Integrated Care data warehouse in NWL.^16^ In 2023, it included more than 2.8 million individuals registered with a general practice (GP) in NWL, around 99% of the NWL patient population.

The primary data were sourced from the SUS_ED table, obtained from the national Secondary Uses Service data,^17^ which includes attendance at urgent and emergency care (UEC) services, including EDs (general and specialised), urgent treatment centres (UTC) and walk-in centres. The table has one row for every time any person attended UEC in NWL or a person registered with a GP in NWL attended a UEC anywhere.

We narrowed our focus to type 1 and 2 EDs because, at the time of this study, there were significant missing data for other UEC types, likely due to recording issues related to the private independent management of several UTCs between 2019 and 2023. The patient key was used to link attendance to primary care data describing the patient’s social and demographic characteristics and chronic conditions based on the Quality of Outcomes Framework (QOF) measures.^18^

### Data and study population

We included the entire population of patients registered with a GP in NWL. Our study observation period was from 5 February 2017 to 30 September 2023, the longest period for which comparable, reliable data were available. We improved the data quality by removing duplicates and same-day entries pertaining to referrals related to initial attendances. Further details are available in the online supplemental material.

### Study timeline

We separated data into three periods: before the COVID-19 pandemic (5 February 2017–1 March 2020); during the pandemic (8 March 2020–13 March 2021) and after the pandemic (14 March 2021–30 September 2023). We defined the end of the pandemic period as the date when COVID-19 governmental lockdown measures began to be eased, according to the Institute for Government timeline.^19^ We used this definition because we sought to account for direct sociopolitical and health system factors that influenced ED attendance. We did not seek to exclude effects of continued circulation of the COVID-19 virus beyond the end of these nationally mandated measures, since COVID-19 represents a component of demand for UEC services, potentially for a substantial period, with no clear endpoint.

### The outcome and explanatory variables

The study outcome variable was the weekly number of all-cause ED attendances. This variable captured changes in the number of ED attendances over time on a granular basis while reducing the effects of daily and weekday-to-weekend fluctuations. Overall weekly ED attendances were then stratified by sociodemographic and health profiles. For demographic characteristics, we used *15-year age groups* and *sex*. We used the quintiles of the English Index of Multiple Deprivation 2019 (IMD19) at the Lower layer Super Output Area level as a socioeconomic status proxy.^20^

We captured the chronic health profile of each attendance using binary variables for 19 long-term QOF conditions (online supplemental table S1). We attached the long-term condition status calculated closest to the attendance date for each attendance. We created two indicators of multimorbidity commonly used in research: multimorbidity and complex multimorbidity.^21^ Based on the WHO definition, multimorbidity was defined as the ‘co-existence of two or more chronic conditions’.^22^ Following Harrison *et al*, we defined complex multimorbidity as having three or more long-term conditions in three different body systems.^23^ We mapped the body systems that the long-term conditions affected (online supplemental table S1) based on the International Classification of Diseases, 10^th^ revision.

### Data analysis and statistical methods

First, we described total ED attendances in the three periods and for each sociodemographic and multimorbidity group. Second, we created time-series data and decomposed it to extract the seasonal effects (eg, winter surges).^23^ Third, to examine if there is a trend in ED presentations, we conducted quasi-Poisson time-series regression models for the weekly counts, in the two periods before and after the pandemic, for all attendances and stratified by sex, age and IMD quintiles. The null hypothesis was that there is no trend after these adjustments. Finally, we analysed weekly attendances for patients with multimorbidity and complex multimorbidity and stratified them by sociodemographic groups (age, sex and IMD19).

We assessed temporal autocorrelation by examining the autocorrelation function (ACF) plots of the model’s Pearson residuals and found significant autocorrelation, particularly lag-1 and lag-3 (ie, dependence on attendance in the previous weeks). The models included a linear term to assess secular trends and covariates for seasonality and lag-1 to lag-4 autocorrelation, consistent with approaches in previous studies.^24^ Quasi-Poisson regression was the best fit for the data, accounting for overdispersion. Missing data were generally below 5%, and further analysis showed the data were missing at random; these were excluded from the stratified analysis for each explanatory variable accordingly. We conducted two sensitivity analyses: one for attendance profiles with smaller age bands and another for the model excluding the winter of 2019–2020 (which exhibited an unusually high peak). Our analysis was at the attendance level and may have included multiple visits by the same person during the study period.

We used SQL Server Management Studio V.18 to retrieve and join data. The data cleaning, analysis and visualisation were conducted in R V.4.2.1. We used the glm function from R’s ‘stats’ package to fit the regression models. The supplementary material presents the sensitivity analysis, the R functions and packages we used in the regression analysis, the ACF plots and the model outputs.

We presented the model’s outcome rate ratios (RRs) and 95% CI annually. We visualised the predicted values with the seasonality and autocorrelation covariates averaged weekly to show the overall attendance trend.

### Patient and public involvement

Patient and public involvement was integral to our research, ensuring its relevance and impact. From the outset, patient representatives contributed to the study design, helping to shape research questions that aligned with patient priorities. They participated in the data access committee. We shared our findings at a learning event organised by the National Institute for Health and Care Research NWL Applied Research Collaboration, which included patients and community representatives. The feedback informed our interpretations of the results.

## Results

**Figure 1:**
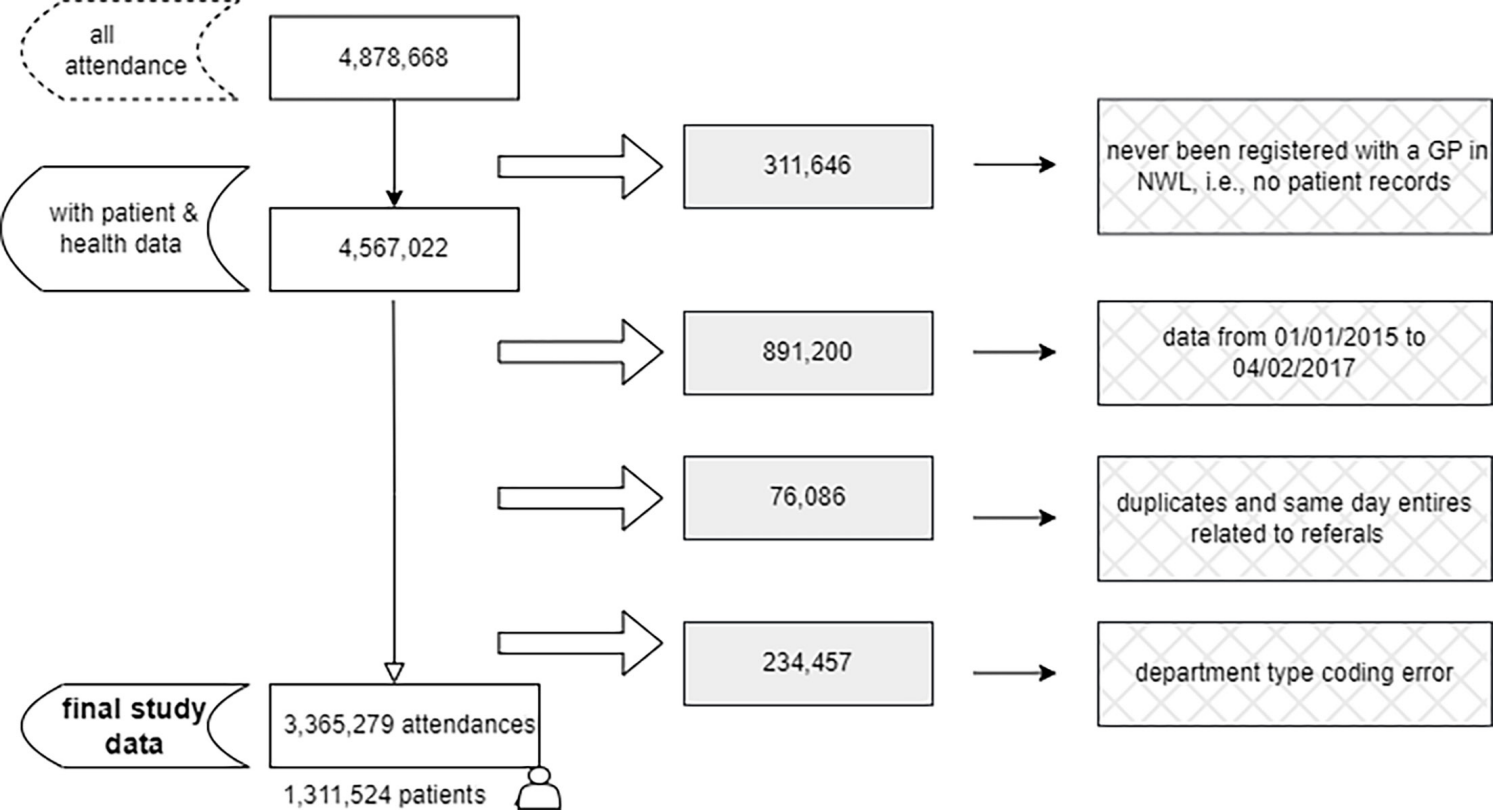
Flow chart for the process toward the final study. GP, general practice; NWL, Northwest London.

We retrieved data on 1 December 2023, for 4 878 668 ED attendances between 1 January 2015 and 30 September 2023. After narrowing the data to our desired study period, applying our inclusion criteria and removing referrals, the final study data included 3 365 279 ED attendances for 1 311,524 patients. Figure 1 shows a flow chart summarising the process leading to the final sample size.

### Descriptive analysis of ED attendance: before, during and after the COVID-19 pandemic

Between 5 February 2017 and 30 September 2023, there were 3 365 279 ED attendances, 50.6% of which were by females (table 1). People aged 31–45 years constituted the highest proportion of attendances (19%), as did people from the second most deprived quintile (31.8%). Overall, 34% (1 144 116) of attendances were by patients with multimorbidity, and 8.4% (282 693) were by patients with complex multimorbidity.

**Table 1:** Sociodemographic and multimorbidity profiles in all ED attendances from February 2017 to September 2023, in the three periods: before, during, and after the COVID-19 pandemic lockdown measures

	Pre-COVID-19 (February 2017–March 2020)		COVID-19 (March 2020–March 2021)		Post COVID-19 (March 2021–September 2023)		Total (February 2017–September 2023)	
	Number	%	Number	%	Number	%	Number	%
Total	**1 596 024**	**47.4**	**406 507**	**12.1**	**1 362 748**	**40**	**3 365 279**	**100**
Female	809 326	50.7	203 083	50.0	691 426	50.7	1 703 835	50.6
Male	775 014	48.6	200 201	49.2	657 488	48.2	1 632 703	48.5
Other	61	0.00	16	0.00	95	0.00	172	0.00
Missing	11 623	0.70	3207	0.70	13 739	1.05	28 569	0.80
0–15	308 160	19.3	51 544	12.7	241 877	17.7	601 581	17.9
16–30	278 913	17.5	70 876	17.4	242 869	17.8	592 658	17.6
31–45	294 990	18.5	83 522	20.5	261 781	19.2	640 293	19.0
46–60	259 339	16.2	75 512	18.6	228 525	16.8	563 376	16.7
61–75	218 313	13.7	60 644	14.9	191 805	14.1	470 762	14.0
76+	225 739	14.1	61 342	15.1	182 499	13.4	469 580	13.9
Missing	10 570	0.66	3067	0.75	13 392	0.98	27 029	0.80
IMD1	227 022	14.2	59 834	14.7	195 001	14.3	481 857	14.3
IMD2	506 690	31.7	129 461	31.9	435 381	31.9	1 071 532	31.8
IMD3	413 394	25.9	104 817	25.8	354 386	26.0	872 597	25.9
IMD4	238 370	14.9	59 281	14.6	199 686	14.7	497 337	14.8
IMD5	110 411	6.92	26 933	6.63	86 128	6.32	223 472	6.65
Missing	100 137	6.27	26 181	6.44	92 166	6.76	218 484	6.50
Multimorbidity	388 083	24.3	119 956	29.5	423 680	31.1	931 719	27.7
Complex	102 340	6.41	31 593	7.77	117 489	8.62	251 422	7.47
Missing	11 626	0.73	3207	0.79	13 740	1.01	28 573	0.85

ED, emergency department; IMD, Index of Multiple Deprivation.

During the period before the COVID-19 pandemic, the age group of 0–15 years accounted for the highest proportion of attendances (19.3%), which reduced considerably during the pandemic (12.7%) and then rose after the pandemic to 17.9%. Conversely, the proportion of attendances for older age groups 61–75 years and 76+ years increased slightly during the pandemic lockdown period from 13.7% to 14.9% and 14.1% to 15.1%, respectively. After the pandemic, attendance by those aged 61–75 years remained at 14.1%, but for those aged 76+ years, it reduced below pre-pandemic levels to 13.4%. Attendances for patients with multimorbidity increased during the pandemic from 24.3% to 29.5% and increased further after the pandemic to 31%. Similarly, attendees with complex multimorbidity rose from 6.4% to 7.8% of attendances during the pandemic and increased further after the pandemic to 8.6%. Attendance patterns by IMD19 and sex remained broadly similar across the three periods (table 1). The table with annual numbers is presented in the online supplemental material S3.

### ED attendance trends

**Figure 2:**
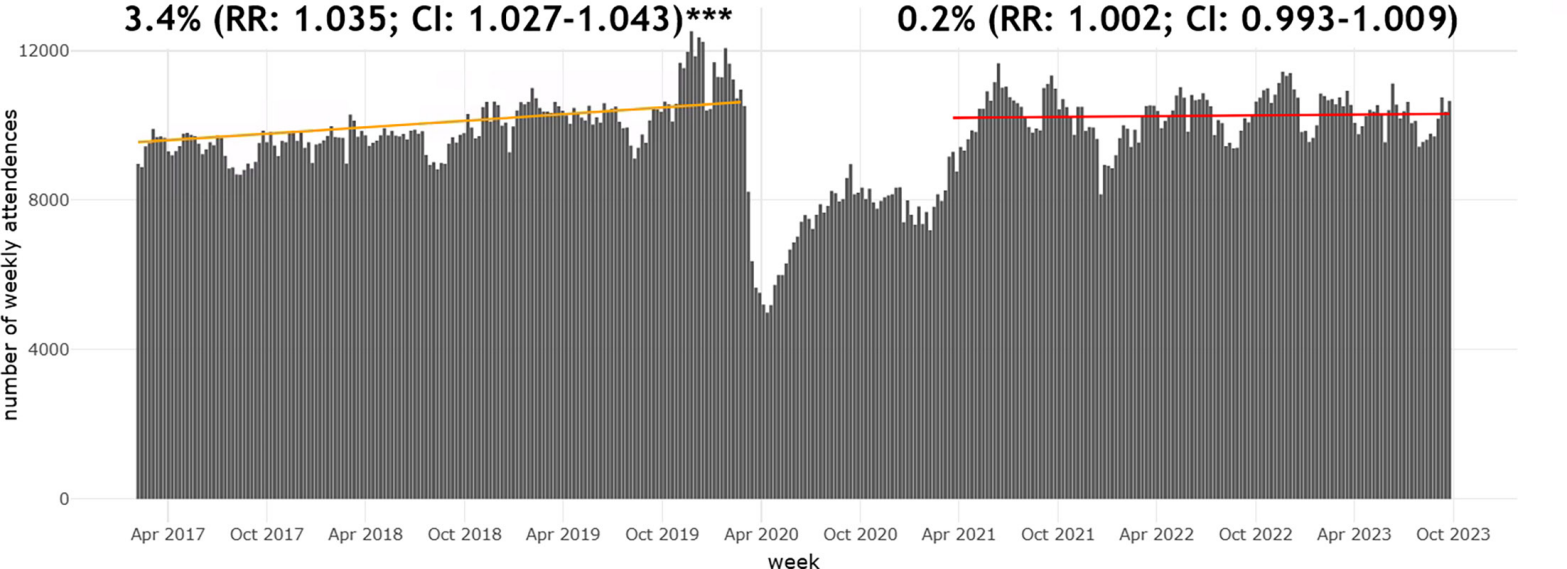
Weekly ED attendance trends from February 2017 to September 2023. Trend lines before and after COVID-19 lockdown measures predicted from the model, with the annual relative rate and their CIs, accounting for seasonality and autocorrelation effects (***p value<0.001). ED, emergency departmeEmergency Department; RR, rate ratio.

Before the pandemic lockdown period, there was a statistically significant rising trend in ED attendance, after accounting for seasonality and autocorrelation effects (figure 2). The annual average increase was 3.4% (RR 1.034; CI 1.026 to 1.042). The highest attendance was in the winter before the pandemic, and the sensitivity analysis showed that this peak did not affect the modelled trend line. The COVID-19 pandemic is represented by the dip in figure 2, corresponding to the lowest attendance levels in our study period. After the pandemic lockdown, the trend flattened with a 0.2% annual increase (RR 1.002; CI 0.993 to 1.009).

### Trends in ED attendance by sociodemographic groups

In the study period before the COVID-19 pandemic, attendance trends for females and males showed an annual RR increase of 1.04 (CI 1.031 to 1.049) and 1.03 (CI 1.023 to 1.038), respectively (table 2), but a nearly flat trend in the period after the pandemic. Attendances increased significantly in all age groups before the pandemic, with the steepest gradient for the youngest group (1.043; CI 1.030 to 1.055). After the pandemic lockdown, trends remained flat for younger age groups, specifically 0–15 years, 16–30 years and 31–45 years. The age group 46–60 years had a slight increase (1.009; CI 1.000 to 1.017). However, like the period before the pandemic, attendances by older age groups 61–75 years and 76+ years demonstrated a statistically significant increasing trend of 1.023 (CI 1.013 to 1.034) and 1.014 (CI 1.003 to 1.024), respectively, although at a slightly lower rate than before the pandemic. Attendances for all IMD19 groups showed statistically significant rising trends before the pandemic, with RR ranging from 1.032 to 1.036. After the pandemic, all plateaued except those from the least deprived (IMD5), which demonstrated a statistically significant decreasing trend with 0.985 RR (CI 0.970 to 0.992) (table 2).

**Table 2:** The annual trend rate ratios and CIs of ED attendance in the study periods before and after the COVID-19 pandemic lockdown measures, by sex, age and IMD19 quintiles, accounting for seasonality and autocorrelation effects

	Pre-COVID-19 (February 2017–March 2020)		Post COVID-19 (March 2021–September 2023)	
	RR	CI	RR	CI
Female	1.040	1.031 to 1.049***	1.001	0.992 to 1.009
Male	1.030	1.023 to 1.038***	1.004	0.995 to 1.014
0–15	1.043	1.030 to 1.055***	0.986	0.966 to 1.005
16–30	1.033	1.023 to 1.044***	0.990	0.979 to 1.002
31–45	1.035	1.024 to 1.047***	1.002	0.993 to 1.011
46–60	1.032	1.030 to 1.053***	1.009	1.000 to 1.017*
61–75	1.032	1.021 to 1.043***	1.023	1.013 to 1.034***
76+	1.033	1.023 to 1.043***	1.014	1.003 to 1.024***
IMD1	1.034	1.024 to 1.045***	0.997	0.987 to 1.006
IMD2	1.036	1.026 to 1.045***	1.006	0.997 to 1.016
IMD3	1.035	1.026 to 1.044***	1.007	0.997 to 1.016
IMD4	1.036	1.026 to 1.046***	1.000	0.988 to 1.008
IMD5	1.032	1.016 to 1.040***	0.981	0.970 to 0.992**

***p value<0.001; **p value<0.01; *p value<0.05.

ED, emergency department; IMD, Index of Multiple Deprivation; RR, rate ratio.

### Attendance by patients with multimorbidity and complex multimorbidity

**Figure 3:**
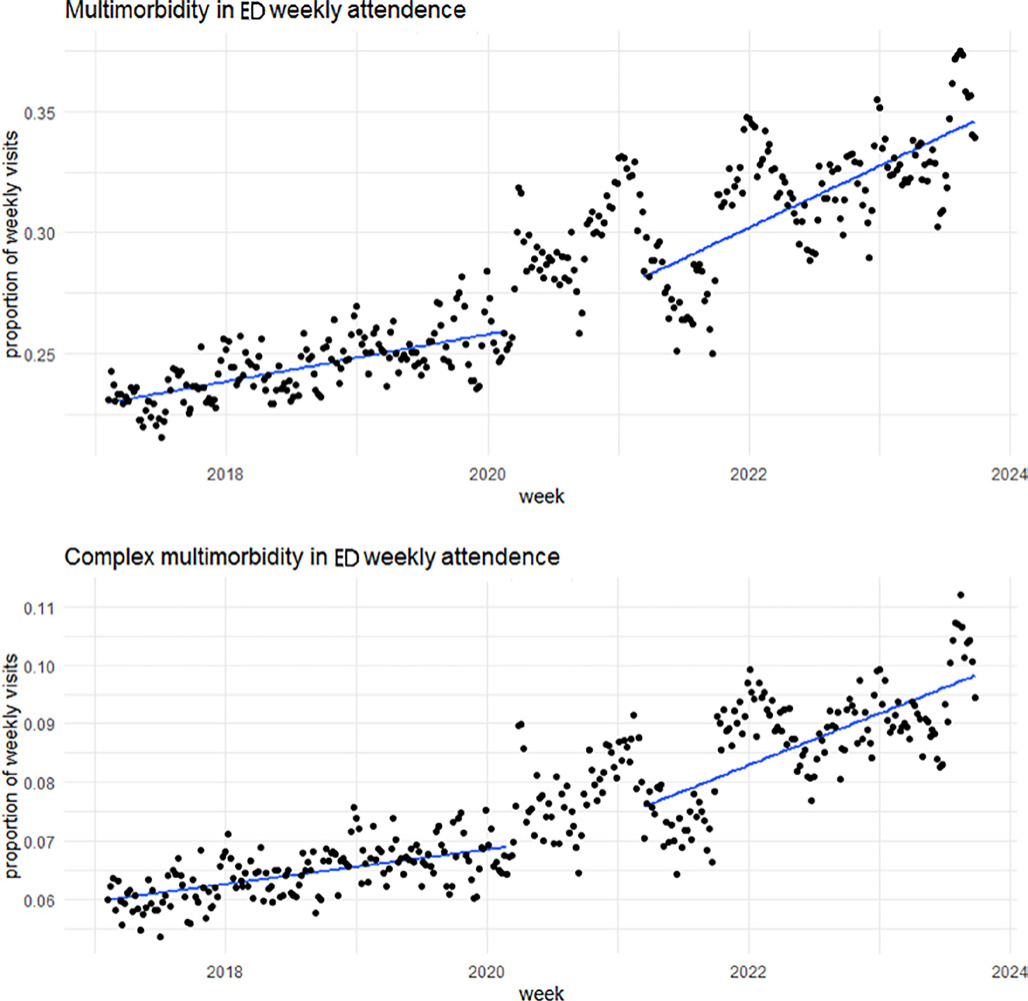
Multimorbidity and complex multimorbidity proportions in weekly ED attendance from February 2017 to September 2023 with trend lines before and after the COVID-19 lockdown measures. ED, emergency departmeEmergency Department.

Figure 3 illustrates a rising trend in the proportion of attendances for patients with multimorbidity and complex multimorbidity prior to the pandemic. The rise became steeper after the COVID-19 pandemic lockdown period ended. The attendance of patients with multimorbidity increased from an average of 24.4% of weekly attendances in the last quarter of 2017 to about 38% in the last quarter of 2023. Similarly, attendance of patients with complex multimorbidity increased from an average of 6.2% to 10.5% in these periods.

As shown in the supplementary material (online supplemental figure S2), female ED attendances had higher proportions of patients with multimorbidity and complex multimorbidity than males, with relatively similar rising gradients. Both multimorbidity and complex multimorbidity increased in all age groups, with the increases being steeper with age, except in the youngest age group (0–15 years). For example, among people aged 61–75 years, the proportion of patients with multimorbidity rose on average from 50% to 72% between the last quarters of 2017 and 2023. At the same time, among those aged 76+ years, the proportion of patient attendances with multimorbidity rose from around 53% to around 80%. Similarly, the proportion of patients with complex multimorbidity almost doubled between the last quarters of 2017 and 2023, from 15% to 28% of weekly attendance. We observed similar increasing trends for the different IMD19 quintiles.

## Discussion

This study examined population-level trends in all-cause ED attendance, highlighting a key issue often overlooked in research and policy: the growing complexity of cases.

Using healthcare records from 2017 to 2023 in NWL, we found that until the COVID-19 pandemic in March 2020, there was a significant upward trend in ED attendance of 3.4% annually. After the COVID-19 public health measures were eased in March 2021, attendance numbers nearly returned to pre-pandemic levels but effectively plateaued after that. However, the increasing trend in the proportion of patient attendances with multimorbidity and complex multimorbidity, which began before the pandemic, almost doubled in the post-pandemic period. By September 2023, the proportion of ED attendances by patients with multimorbidity and complex multimorbidity reached more than 42% and 11%, respectively. The proportional increase was most notable for older people.

There were also changes in the demographic profiles of ED attendance. Before the pandemic, trends for all demographic and socioeconomic groups were rising. After the pandemic, trends for all groups stabilised except for older age groups (61–75 years and 76+ years), which continued in a statistically significant increasing trend, although at a lower rate than before the pandemic, and to a lesser extent in the age group 41–60 years. In contrast, the ED attendance trend decreased for the least deprived.

Similar overall trends and patterns in ED attendance have been documented in the UK.^3 12 13 15 25^ However, none of these sources investigated multimorbidity patterns and sociodemographic profiles. Before the pandemic, between 2010 and 2015, the UK national figure for the annual increase rate in ED attendance was 1.8%.^26^ This was lower than our finding, but the prior study used a longer temporal resolution than our study and was not adjusted. Our findings demonstrated noticeable winter surges each year throughout the study period, related to increased incidence of respiratory conditions, particularly in the winter of 2019/2020 before the pandemic.^27^

The COVID-19 pandemic forced rapid structural and behavioural responses in how health services were delivered and accessed. These changes offer one possible explanation for our findings on ED demand.^13^ For example, there were changes and improvements in the structure of primary care and integration between care pathways^11^; there was also operational expansion and promotion of technology-enabled healthcare services for remote consultations and triage.^3 10^ Furthermore, there has been a greater focus on developing urgent care centres where patients with less complex urgent or emergency care needs can attend or be streamed from the hospital’s front door. These arrangements were designed to reduce avoidable attendance during the pandemic, but some of them and their effects may have persisted to some extent afterwards.^28^ This might explain our finding that younger people’s attendance plateaued after the pandemic. Prior to the pandemic, younger and healthier people were a major source of increases in attendance, but they are also thought to have had most of the avoidable attendances.^11^ Additionally, more advantaged populations might have derived greater benefit from such developments, which could explain our finding that their trend in attendance decreased slightly after the pandemic.^10^

However, although the growth rate in the number of people attending ED has stabilised after the pandemic, this does not imply less pressure on services.^3 5^ As we found, multimorbidity and complex multimorbidity are increasing in ED attendances, which are known to be among the strongest clinical predictors of ED attendance and longer hospital stays.^29^ Therefore, it may not be surprising that the other reported ED services’ performance indicators (such as waiting time) look similar or worse than before the pandemic,^3^ because the system provides care for patients who, on average, have more complex care needs.

Several factors could be behind the increasing multimorbidity in ED, over and above the long-term increase in prevalence in the population.^9^ First, delayed diagnosis and health support for patients with multimorbidity during the pandemic could have increased their attendance following the easing of lockdown measures.^11^ Second, it has been shown that patients with multimorbidity use the ED more than other services because of convenience and trust, or because other pathways do not meet their needs.^8^ The numerous challenges facing the English community and social care system, as well as the increase in unmet social care needs over the last decade,^30 31^ may have led to a further rise in demand for ED services among patients with multimorbidity.^9^ For example, a report by Age UK in 2023 showed that between 2016 and 2022, the percentage of older people in the UK who felt supported in managing their chronic health problems declined by 20%.^31^ Third, the ongoing circulation of the COVID-19 virus and the development of long COVID are known to exacerbate pre-existing long-term conditions,^32^ contributing to increased ED attendance.

While all these factors may contribute to explaining our findings, they were out of the scope of our analysis. Further research is required to study not only the causes of the observed increases in multimorbidity in EDs, but also to understand what policies and interventions are needed to ensure that UEC systems can meet the care needs of the populations they serve.

### Strengths and limitations

This study provides a longitudinal time-series analysis of ED attendance trends and sociodemographic and multimorbidity profiles in a large population, covering periods before, during and after the COVID-19 pandemic. A strength of our research was the extensive linked primary and secondary care data, which were highly representative of the NWL patient population and included all patients registered with NWL GPs. The diversity of the NWL population strengthens the generalisability to other urban areas,^16^ although it may limit generalisability to rural areas with an older population. We ensured the quality of the data and used models that best fit it, taking into account autocorrelation.

This observational study is limited in its ability to establish causality and identify specific reasons for the observed results. We did not account for crowding, which might influence attendance profiles. Excluding urgent centres (types 3 and 4) limited our understanding of their role in stabilising ED trends after the pandemic. Since our study period ended on 30 September 2023, more recent trends were not included. Although the data regarding chronic conditions in our study were from primary care records, they have some limitations. We were restricted to 19 QOF measures; however, these were the most common conditions. There might have been coding differences, which could restrict their comparability across time and with other studies. However, data were regularly monitored and updated, and the reasonable study length without major updates for QOF measures or guidelines reduced recording problems.^18^ Using the governmental lockdown timeline did not reflect the COVID-19 virus distribution, and we did not account for changes in IMD19, which were out of the scope of our data.

### Conclusion and recommendations

This study highlights shifts in ED use after the COVID-19 pandemic and provides insights into longer-term population care needs. These findings are of importance to ED clinicians and managers, as well as to planners and policymakers with wider health system leadership roles.

Increasing multimorbidity is putting pressure on UEC systems internationally. Reducing such pressure will depend on understanding and mitigating the trends illustrated in this study. Evaluation of the rapid changes prompted by the pandemic may present learning opportunities applicable in the long term.

## Supplementary material

10.1136/emermed-2024-214412 online supplemental file 1

10.1136/emermed-2024-214412 online supplemental file 2

## Data Availability

Data may be obtained from a third party and are not publicly available.
