# Supplementary material for: Shifting patterns in emergency department attendance: a time series analysis
Source: Emerg Med J. 2025 Dec 4;43(4):e214412. doi: 10.1136/emermed-2024-214412 (PMC13151452; doi:10.1136/emermed-2024-214412)
Supplement: online supplemental file 1 [file emermed-43-4-s001.docx]

# **Supplementary material**

**Data quality improvement**

We improved the quality of the dataset by three actions. First, we removed attendances for the same patient on the same date and arrival hour, and second, we removed attendances when there were more than two attendances for the same patient on the same date. Most of these rows did not appear to reflect actual ED attendances and were mainly redundant attendances, probably related to the same site referrals (i.e. other departments) or different sites. Instead of continuing with the same SPELL (attendance), a new SPELL was generated at the referred departments/sites, mainly for financial administration purposes in these sites/departments, as all columns were mostly identical for these duplicated rows, except the final tariff column. In the first case, we implemented an algorithm that discarded duplicate rows while keeping the attendance for the longest duration; in the second, we kept attendance for the highest total tariffs. Finally, we removed attendances that were wrongly coded in the system as attendances to an ED site, but after investigation with the data managers, it was, according to the NHS definitions, an urgent treatment centre.

Table S1: Long-term conditions and the mapped body system

| **Condition** | **System** | **Condition** | **System** |
| --- | --- | --- | --- |
| Asthma | Respiratory | Chronic Kidney Disease | Genitourinary |
| COPD | Respiratory | Epilepsy | Nervous |
| Dementia | Nervous | Heart failure | Circulatory |
| Osteoporosis | Musculoskeletal | Severe Mental health conditions | Mental and behavioural |
| Ischaemic heart disease | Circulatory | Stroke and TIA (Transient Ischaemic Attack) | Circulatory |
| Atrial Fibrillation | Circulatory disorders | Obesity | Endocrine, nutrition and metabolic |
| Depression | Mental and behavioural | Cancer | Neoplasms |
| Hypertension | Circulatory disorders | Rheumatoid arthritis | Musculoskeletal |
| Peripheral Arterial Disease | Circulatory disorders | Coronary heart disease | Circulatory disorders |
| Diabetes | Endocrine, nutrition and metabolic |  |  |

**Table S2: R packages and functions used in the regression analysis**

| **Functions** | **R Package** | **Use** |
| --- | --- | --- |
| ts | stats | To create time series data |
| Isoweek | lubridate | To get the week number |
| glm(, family=”quasipossion) | stats | Quasi Poisson regression model |
| acf(residuals(model, type=”pearson”)) | stats | Autocorrelation Function (ACF) plot |
| exp(confit()); | stats | Get the rate ratios and their confidence intervals |
| predict() | stats | Generate predicted values |
| ggplot() | ggplot2 | To plot the predicted values |

**Model’s output before the pandemic (pre_ts= number of attendance weekly, n=week number)**

**
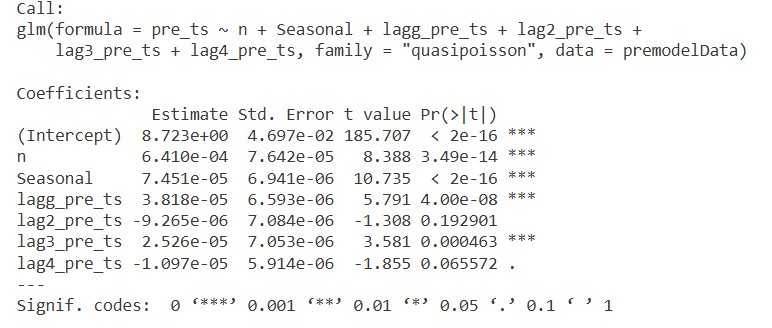
**

**Model’s output after the pandemic (pre_ts= number of attendance weekly, n=week number)**

**
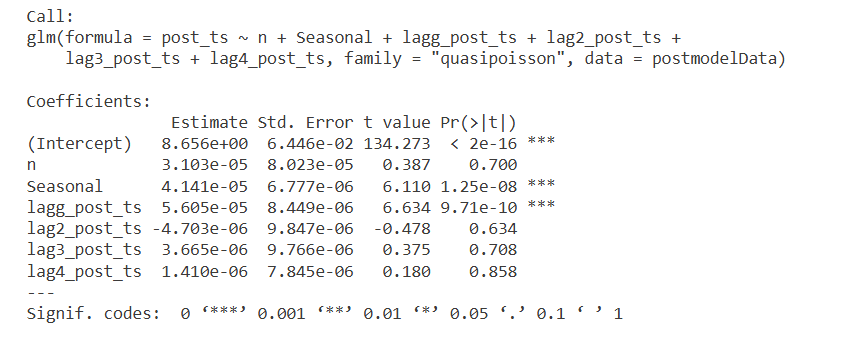
**

**Figure S2: Autocorrelation Function ACF plots of Pearson residuals** (The x axis is lag-1 to lag-20, and below the blue line is acceptable autocorrelation)

**The model for the period before the COVID-19 pandemic**


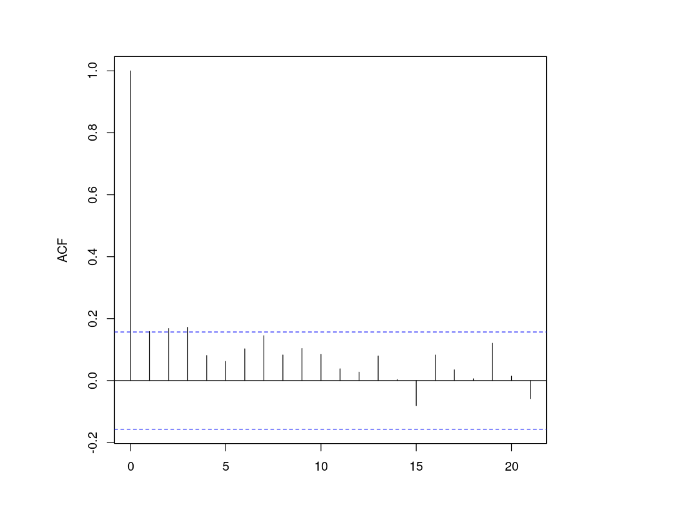


**The model for the period after the COVID-19 pandemic**


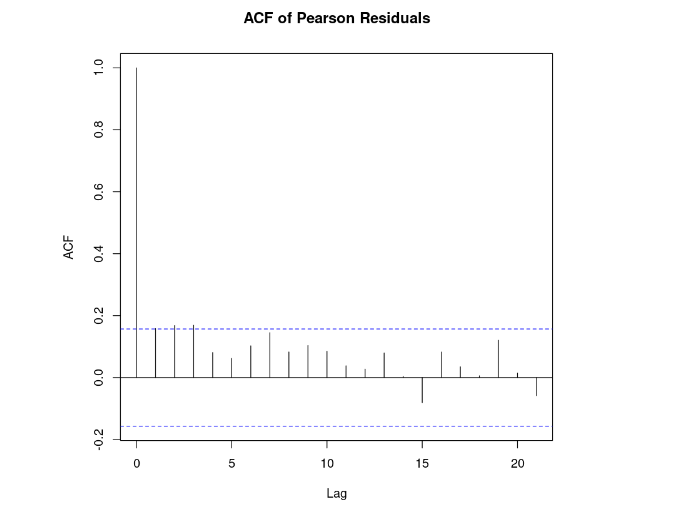


**Sensitivity and missing data analysis**

We did a sensitivity analysis for ED attendance with smaller 10-year age groups, and it did not show different patterns or unusual results compared to 15-year age groups. We also analysed patterns of ED attendance only for ages between 0-2, and it also depicted similar trends as 0-15. We did a missing data analysis and plotted the patterns of missing data in all the explanatory variables. The results did not show any pattern to suspect that the missing data could have a negative impact on the data integrity.

We did a sensitivity analysis to test the model trend line for the period before the COVID-19 pandemic, without the last 16 weeks, to assess if the large increase in attendance in the winter of 2019-2020 impacted the RR estimated by the model and its statistical significance. We found that this increase in attendance has no effect. The model output for the model without the last 16 weeks is shown below.

Model’s output before the pandemic, without the last 16 weeks (pre_ts= number of weekly attendance, n=week number)


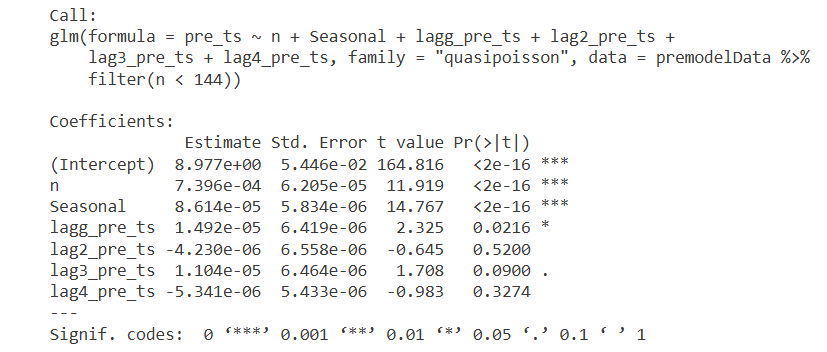


Yearly RR = exp(0.00074)^52 = =3.9%

| 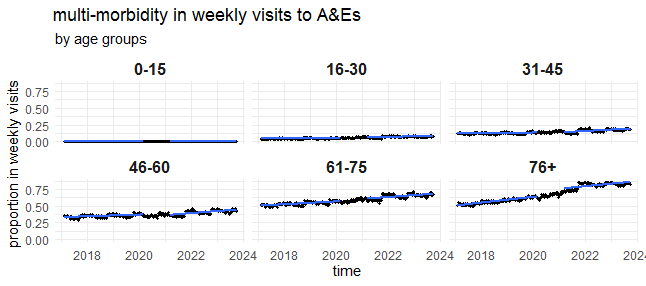 | 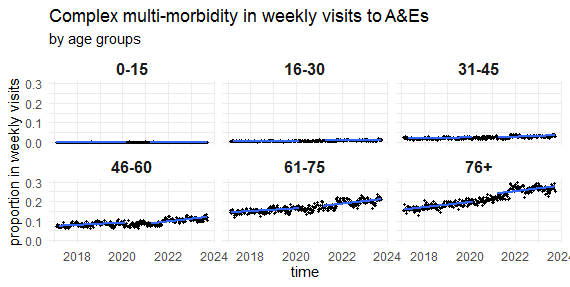 |
| --- | --- |
| 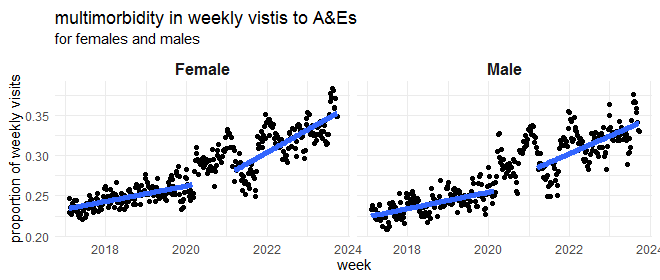 | 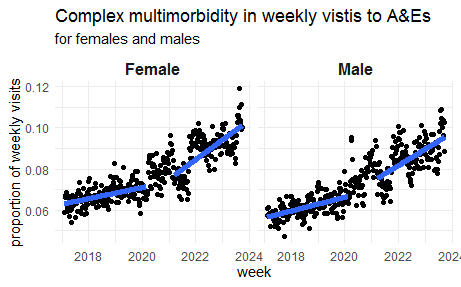 |
| 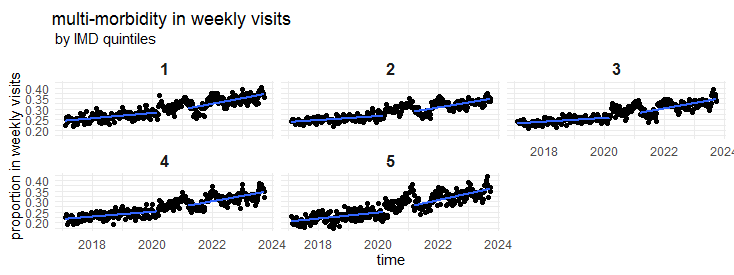 | 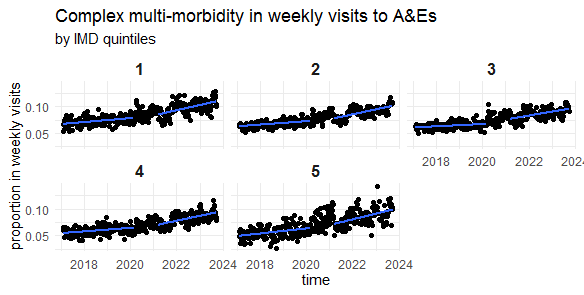 |
| Figure S3: Multimorbidity and complex multimorbidity trends in ED attendance by age, sex and IMD19 | |

**Multimorbidity and complex multimorbidity by the sociodemographic groups in ED attendance**
