# Supplementary material for: Shifting patterns in emergency department attendance: a time series analysis
Source: Emerg Med J. 2025 Dec 4;43(4):e214412. doi: 10.1136/emermed-2024-214412 (PMC13151452; doi:10.1136/emermed-2024-214412)
Supplement: online supplemental file 2 [file emermed-43-4-s002.pdf]

Table S3: Sociodemographic and multimorbidity profiles in all ED attendances from February 2017 to September 2023, with total and annual numbers in the three periods: before, during, and after the COVID-19 pandemic lockdown measures.

	**pre COVID-19** (Feb 2017-Mar 2020)			**COVID-19 Lockdown** (Mar 2020-Mar 2021)			**post COVID-19** (Mar 2021-Sep 2023)			**Total** (Feb 2017-Sep 2023)	
	**Number**	**Annual number**	**%**	**Number**	**Annual number**	**%**	**Number**	**Annual number**	**%**	**Number**	**%**
**Total**	**1,596,024**	**519,956**	**47.4**	**406,507**	**402,483**	**12.1**	**1,362,748**	**534,421**	**40**	**3365279**	**100**
Female	809,326	263,641	50.7	203,083	200,073	50.0	691,426	271,158	50.7	1703835	50.6
Male	775,014	252,363	48.6	200,201	198,218	49.2	657,488	257,642	48.2	1632703	48.5
Other	61	20	0.00	16	16	0.00	95	37	0.00	172	0.00
missing	11,623	3,785	0.70	3,207	3,174	0.70	13,739	5,390	1.05	28569	0.80
0-15	308,160	100,332	19.3	51,544	51,030	12.7	241,877	94,862	17.7	601581	17.9
16-30	278,913	90,856	17.5	70,876	70,171	17.4	242,869	95,255	17.8	592658	17.6
31-45	294,990	96,061	18.5	83,522	82,697	20.5	261,781	102,669	19.2	640293	19.0
46-60	259,339	84,519	16.2	75,512	74,772	18.6	228,525	89,527	16.8	563376	16.7
61-75	218,313	71,094	13.7	60,644	60,038	14.9	191,805	75,098	14.1	470762	14.0
76+	225,739	73,486	14.1	61,342	60,739	15.1	182,499	71,568	13.4	469580	13.9
Missing	10,570	3,445	0.66	3,067	3,036	0.75	13,392	5,253	0.98	27029	0.80
IMD1	227,022	73,997	14.2	59,834	59,237	14.7	195,001	76,471	14.3	481857	14.3
IMD2	506,690	165,073	31.7	129,461	128,178	31.9	435,381	170,767	31.9	1071532	31.8
IMD3	413,394	134,675	25.9	104,817	103,779	25.8	354,386	139,019	26.0	872597	25.9
IMD4	238,370	77,672	14.9	59,281	58,690	14.6	199,686	78,346	14.7	497337	14.8
IMD5	110,411	35,942	6.92	26,933	26,926	6.63	86,128	33,777	6.32	223472	6.65
Missing	100,137	32,618	6.27	26,181	25,6783	6.44	92,166	36,148	6.76	218484	6.50
Multimorbidity	388,083	126,405	24.3	119,956	118,773	29.5	423,680	166,130	31.1	931719	27.7
Complex	102,340	33,359	6.41	31,593	31,277	7.77	117,489	46,087	8.62	251422	7.47
Missing	11,626	3,788	0.73	3,207	3,174	0.79	13,740	5,390	1.01	28573	0.85
